# Supplementary material for: Attentional amplification of neural codes for number independent of other quantities along the dorsal visual stream
Source: eLife. 2019 Jul 24;8:e45160. doi: 10.7554/eLife.45160 (PMC6693892; doi:10.7554/eLife.45160)
Supplement: Supplementary file 1. — The table reports t-values, degrees of freedom (Dof), p-values and confidence intervals of the two-tailed t-tests against 0.5 (chance level) used to evaluate the accuracies of number classification for every ROI and task. [file elife-45160-supp1.docx]

Supplementary File 1.

|  | Task: Judge Number | | | | |  | Task: Judge Size | | | | |
| --- | --- | --- | --- | --- | --- | --- | --- | --- | --- | --- | --- |
| ROI\stats | t-val | Dof | p-val | CI (95%) | |  | t-val | Dof | p-val | CI (95%) | |
| V1-3 | 8.22 | 19 | <10^-6^ | 0.60 | 0.67 |  | 9.14 | 19 | <10^-6^ | 0.59 | 0.65 |
| V3AB-V7 | 10.59 | 19 | <10^-6^ | 0.63 | 0.70 |  | 7.85 | 19 | <10^-6^ | 0.58 | 0.64 |
| IPS 1-5 | 9.58 | 19 | <10^-6^ | 0.64 | 0.71 |  | 6.10 | 19 | 0.000007 | 0.57 | 0.64 |
| V1 | 9.26 | 19 | <10^-6^ | 0.59 | 0.65 |  | 6.76 | 19 | 0.000002 | 0.57 | 0.64 |
| V2 | 7.57 | 19 | <10^-6^ | 0.59 | 0.65 |  | 7.71 | 19 | <10^-6^ | 0.58 | 0.65 |
| V3 | 9.81 | 19 | <10^-6^ | 0.59 | 0.64 |  | 7.13 | 19 | 0.000001 | 0.57 | 0.64 |
| V3AB | 10.09 | 19 | <10^-6^ | 0.61 | 0.67 |  | 5.05 | 19 | 0.00007 | 0.55 | 0.61 |
| V7 | 9.71 | 19 | <10^-6^ | 0.62 | 0.68 |  | 5.93 | 19 | 0.00001 | 0.55 | 0.60 |
| IPS12 | 8.57 | 19 | <10^-6^ | 0.63 | 0.71 |  | 6.78 | 19 | 0.000002 | 0.56 | 0.62 |
| IPS345 | 8.92 | 19 | <10^-6^ | 0.63 | 0.71 |  | 6.57 | 19 | 0.000003 | 0.55 | 0.61 |
