# Supplementary material for: Attentional amplification of neural codes for number independent of other quantities along the dorsal visual stream
Source: eLife. 2019 Jul 24;8:e45160. doi: 10.7554/eLife.45160 (PMC6693892; doi:10.7554/eLife.45160)
Supplement: Supplementary file 2. — The table shows t-values, degrees of freedom (Dof), p-values and confidence intervals of two-tailed t-tests against zero across subjects for every ROI and dimension (N: number, S: average item size, TFA: total field area, TSA: total surface area, D: density) for the number (left table) and size (right table) tasks. [file elife-45160-supp2.docx]

Supplementary File 2.

| Number (N) | | | | | | | | | | | |
| --- | --- | --- | --- | --- | --- | --- | --- | --- | --- | --- | --- |
|  | Task: Judge Number | | | | |  | Task: Judge Size | | | | |
| ROI\stats | t-val | Dof | p-val | CI (95%) | |  | t-val | Dof | p-val | CI (95%) | |
| V1-3 | 7.87 | 19 | <10^-6^ | 0.17 | 0.29 |  | 4.08 | 19 | 0.0006 | 0.06 | 0.19 |
| V3AB-V7 | 8.42 | 19 | <10^-6^ | 0.25 | 0.42 |  | 3.94 | 19 | 0.0009 | 0.05 | 0.15 |
| IPS 1-5 | 6.90 | 19 | 0.000001 | 0.21 | 0.40 |  | 3.39 | 19 | 0.003 | 0.03 | 0.13 |
| V1 | 5.99 | 19 | 0.000009 | 0.11 | 0.22 |  | 2.45 | 19 | 0.024 | 0.01 | 0.15 |
| V2 | 6.48 | 19 | 0.000003 | 0.14 | 0.27 |  | 4.52 | 19 | 0.0002 | 0.08 | 0.22 |
| V3 | 6.28 | 19 | 0.000005 | 0.16 | 0.32 |  | 3.74 | 19 | 0.001 | 0.05 | 0.16 |
| V3AB | 7.06 | 19 | 0.000001 | 0.17 | 0.31 |  | 2.54 | 19 | 0.020 | 0.01 | 0.11 |
| V7 | 6.68 | 19 | 0.000002 | 0.20 | 0.39 |  | 2.88 | 19 | 0.010 | 0.02 | 0.11 |
| IPS12 | 7.19 | 19 | 0.000001 | 0.22 | 0.40 |  | 2.75 | 19 | 0.013 | 0.02 | 0.12 |
| IPS345 | 5.75 | 19 | 0.00002 | 0.17 | 0.36 |  | 3.19 | 19 | 0.005 | 0.02 | 0.11 |
| Average Item Size (S) | | | | | | | | | | | |
|  | Task: Judge Number | | | | |  | Task: Judge Size | | | | |
| ROI\stats | t-val | Dof | p-val | CI (95%) | |  | t-val | Dof | p-val | CI (95%) | |
| V1-3 | 0.78 | 19 | 0.447 | -0.02 | 0.04 |  | -0.85 | 19 | 0.405 | -0.03 | 0.01 |
| V3AB-V7 | 0.04 | 19 | 0.972 | -0.04 | 0.04 |  | 1.81 | 19 | 0.086 | 0.00 | 0.06 |
| IPS 1-5 | 0.55 | 19 | 0.587 | -0.03 | 0.05 |  | 1.83 | 19 | 0.083 | -0.01 | 0.10 |
| V1 | -0.07 | 19 | 0.946 | -0.04 | 0.03 |  | -0.29 | 19 | 0.777 | -0.03 | 0.03 |
| V2 | 0.01 | 19 | 0.996 | -0.03 | 0.03 |  | -1.82 | 19 | 0.085 | -0.04 | 0.00 |
| V3 | 0.70 | 19 | 0.495 | -0.02 | 0.05 |  | -0.98 | 19 | 0.339 | -0.03 | 0.01 |
| V3AB | 0.21 | 19 | 0.836 | -0.03 | 0.04 |  | 0.76 | 19 | 0.459 | -0.02 | 0.03 |
| V7 | 0.23 | 19 | 0.818 | -0.04 | 0.05 |  | 1.21 | 19 | 0.243 | -0.02 | 0.07 |
| IPS12 | 0.69 | 19 | 0.500 | -0.03 | 0.05 |  | 1.63 | 19 | 0.119 | -0.01 | 0.10 |
| IPS345 | 0.55 | 19 | 0.587 | -0.02 | 0.04 |  | 1.48 | 19 | 0.155 | -0.01 | 0.08 |
| Total Field Area (TFA) | | | | | | | | | | | |
|  | Task: Judge Number | | | | |  | Task: Judge Size | | | | |
| ROI\stats | t-val | Dof | p-val | CI (95%) | |  | t-val | Dof | p-val | CI (95%) | |
| V1-3 | 7.33 | 19 | 0.000001 | 0.22 | 0.39 |  | 5.37 | 19 | 0.00003 | 0.18 | 0.41 |
| V3AB-V7 | 4.16 | 19 | 0.0005 | 0.06 | 0.17 |  | 2.77 | 19 | 0.012 | 0.03 | 0.18 |
| IPS 1-5 | 1.47 | 19 | 0.157 | -0.02 | 0.09 |  | 0.78 | 19 | 0.445 | -0.04 | 0.08 |
| V1 | 7.33 | 19 | 0.000001 | 0.18 | 0.32 |  | 6.00 | 19 | 0.000009 | 0.16 | 0.34 |
| V2 | 7.09 | 19 | 0.000001 | 0.23 | 0.42 |  | 5.87 | 19 | 0.00001 | 0.19 | 0.40 |
| V3 | 6.77 | 19 | 0.000002 | 0.19 | 0.36 |  | 5.49 | 19 | 0.00003 | 0.18 | 0.41 |
| V3AB | 5.17 | 19 | 0.00006 | 0.09 | 0.20 |  | 3.57 | 19 | 0.002 | 0.06 | 0.22 |
| V7 | 2.43 | 19 | 0.025 | 0.01 | 0.11 |  | 1.43 | 19 | 0.170 | -0.02 | 0.11 |
| IPS12 | 1.38 | 19 | 0.185 | -0.02 | 0.09 |  | 0.83 | 19 | 0.419 | -0.03 | 0.07 |
| IPS345 | 0.88 | 19 | 0.392 | -0.03 | 0.08 |  | -0.33 | 19 | 0.747 | -0.06 | 0.04 |
| Total Surface Area (TSA) | | | | | | | | | | | |
|  | Task: Judge Number | | | | |  | Task: Judge Size | | | | |
| ROI\stats | t-val | Dof | p-val | CI (95%) | |  | t-val | Dof | p-val | CI (95%) | |
| V1-3 | 3.02 | 19 | 0.007 | 0.02 | 0.11 |  | 4.33 | 19 | 0.0004 | 0.04 | 0.11 |
| V3AB-V7 | -0.24 | 19 | 0.815 | -0.05 | 0.04 |  | 1.22 | 19 | 0.238 | -0.02 | 0.06 |
| IPS 1-5 | -2.65 | 19 | 0.016 | -0.08 | -0.01 |  | 0.79 | 19 | 0.439 | -0.03 | 0.07 |
| V1 | 2.71 | 19 | 0.014 | 0.01 | 0.10 |  | 3.34 | 19 | 0.003 | 0.02 | 0.08 |
| V2 | 3.21 | 19 | 0.005 | 0.02 | 0.11 |  | 4.07 | 19 | 0.0007 | 0.03 | 0.11 |
| V3 | 2.42 | 19 | 0.026 | 0.01 | 0.09 |  | 2.61 | 19 | 0.017 | 0.01 | 0.08 |
| V3AB | 0.56 | 19 | 0.580 | -0.03 | 0.05 |  | 0.94 | 19 | 0.361 | -0.02 | 0.05 |
| V7 | -1.13 | 19 | 0.271 | -0.07 | 0.02 |  | 0.50 | 19 | 0.622 | -0.03 | 0.05 |
| IPS12 | -2.52 | 19 | 0.021 | -0.09 | -0.01 |  | 0.95 | 19 | 0.352 | -0.02 | 0.07 |
| IPS345 | -3.15 | 19 | 0.005 | -0.08 | -0.02 |  | 0.10 | 19 | 0.921 | -0.05 | 0.05 |
| Density (D) | | | | | | | | | | | |
|  | Task: Judge Number | | | | |  | Task: Judge Size | | | | |
| ROI\stats | t-val | Dof | p-val | CI (95%) | |  | t-val | Dof | p-val | CI (95%) | |
| V1-3 | 2.07 | 19 | 0.052 | 0.0007 | 0.14 |  | 2.44 | 19 | 0.025 | 0.01 | 0.09 |
| V3AB-V7 | -0.76 | 19 | 0.456 | -0.08 | 0.04 |  | 1.00 | 19 | 0.328 | -0.03 | 0.08 |
| IPS 1-5 | 1.50 | 19 | 0.151 | -0.02 | 0.10 |  | 3.30 | 19 | 0.004 | 0.03 | 0.12 |
| V1 | 3.64 | 19 | 0.002 | 0.04 | 0.16 |  | 3.23 | 19 | 0.004 | 0.03 | 0.13 |
| V2 | 2.17 | 19 | 0.043 | 0.00 | 0.14 |  | 1.60 | 19 | 0.127 | -0.01 | 0.10 |
| V3 | 0.75 | 19 | 0.464 | -0.04 | 0.09 |  | 0.96 | 19 | 0.348 | -0.03 | 0.07 |
| V3AB | -0.98 | 19 | 0.339 | -0.07 | 0.02 |  | -0.23 | 19 | 0.821 | -0.05 | 0.04 |
| V7 | 0.00 | 19 | 0.998 | -0.05 | 0.05 |  | 1.83 | 19 | 0.083 | -0.01 | 0.10 |
| IPS12 | 1.33 | 19 | 0.200 | -0.02 | 0.09 |  | 3.04 | 19 | 0.007 | 0.02 | 0.12 |
| IPS345 | 1.88 | 19 | 0.075 | -0.004 | 0.08 |  | 2.90 | 19 | 0.009 | 0.02 | 0.10 |
