# Supplementary material for: Attentional amplification of neural codes for number independent of other quantities along the dorsal visual stream
Source: eLife. 2019 Jul 24;8:e45160. doi: 10.7554/eLife.45160 (PMC6693892; doi:10.7554/eLife.45160)
Supplement: Supplementary file 3. — The table reports t-values, degrees of freedom (Dof), p-values and confidence intervals of the two-tailed t-tests against 0.5 (chance level) used to evaluate the significance of task classification for every ROI. [file elife-45160-supp3.docx]

Supplementary File 3.

|  | Decode Task | | | | |
| --- | --- | --- | --- | --- | --- |
| ROI\stats | t-val | Dof | p-val | CI (95%) | |
| V1-3 | 1.35 | 19 | 0.192 | .49 | .55 |
| V3AB-V7 | 3.74 | 19 | 0.001 | .54 | .64 |
| IPS 1-5 | 2.79 | 19 | 0.012 | .52 | .65 |
| V1 | -.51 | 19 | 0.615 | .46 | .52 |
| V2 | .69 | 19 | 0.597 | .48 | .54 |
| V3 | .73 | 19 | 0.474 | .48 | .54 |
| V3AB | 2.98 | 19 | 0.007 | .52 | .61 |
| V7 | 4.26 | 19 | 0.0004 | .55 | .64 |
| IPS12 | 3.79 | 19 | 0.001 | .55 | .67 |
| IPS345 | 2.13 | 19 | 0.046 | .50 | .60 |
