# Supplementary material for: Attentional amplification of neural codes for number independent of other quantities along the dorsal visual stream
Source: eLife. 2019 Jul 24;8:e45160. doi: 10.7554/eLife.45160 (PMC6693892; doi:10.7554/eLife.45160)
Supplement: Supplementary file 4. — (a) Statistical results for the performance of the classifiers trained to discriminate between numerosities during the number (left table) and size (right table) task for the ROI IPS excluding IPS 0–5. The table reports the statistical results of the two-tailed t-tests against 0.5 (chance level). (b). Statistical results for the performance of the classifiers trained to discriminate between tasks for the ROI IPS excluding IPS 0–5. The table reports the statistical results of the two-tailed t-tests against 0.5 (chance level). (c) Statistical results for beta weights obtained from the RSA multiple regression for the ROI IPS excluding IPS 0–5. The table shows t-values, degrees of freedom (Dof), p-values and confidence intervals of two-tailed t-tests against zero across subjects for every dimension (N: number, S: average item size, TFA: total field area, TSA: total surface area, D: density) for the number (left table) and size (right table) tasks. [file elife-45160-supp4.docx]

Supplementary File 4a

|  | Task: Judge Number | | | | |  | Task: Judge Size | | | | |
| --- | --- | --- | --- | --- | --- | --- | --- | --- | --- | --- | --- |
| ROI\stats | t-val | Dof | p-val | CI (95%) | |  | t-val | Dof | p-val | CI (95%) | |
| IPS excl IPS 0-5 | 8.94 | 19 | <10^-6^ | 0.63 | 0.70 |  | 5.87 | 19 | 0.000001 | 0.55 | 0.60 |

Supplementary File 4b

|  | Task | | | | |
| --- | --- | --- | --- | --- | --- |
| ROI\stats | t-val | Dof | p-val | CI (95%) | |
| IPS excl IPS 0-5 | 4.29 | 19 | 0.0004 | 0.56 | 0.67 |

Supplementary File 4c

| IPS excl IPS 0-5 | | | | | | | | | | | |
| --- | --- | --- | --- | --- | --- | --- | --- | --- | --- | --- | --- |
|  | Task: Judge Number | | | | |  | Task: Judge Size | | | | |
| ROI\stats | t-val | Dof | p-val | CI (95%) | |  | t-val | Dof | p-val | CI (95%) | |
| N | 5.74 | 19 | 0.00001 | 0.15 | 0.32 |  | 1.78 | 19 | 0.091 | -0.01 | 0.08 |
| S | -0.73 | 19 | 0.475 | -0.06 | 0.03 |  | 1.89 | 19 | 0.074 | 0 | 0.09 |
| TSA | -0.17 | 19 | 0.864 | -0.05 | 0.04 |  | -1.94 | 19 | 0.068 | -0.06 | 0 |
| TFA | -1.62 | 19 | 0.121 | -0.08 | 0.01 |  | 0.56 | 19 | 0.581 | -0.03 | 0.05 |
| Dens | -0.32 | 19 | 0.756 | -0.05 | 0.04 |  | 0.62 | 19 | 0.544 | -0.03 | 0.05 |
