# Supplementary material for: Attentional amplification of neural codes for number independent of other quantities along the dorsal visual stream
Source: eLife. 2019 Jul 24;8:e45160. doi: 10.7554/eLife.45160 (PMC6693892; doi:10.7554/eLife.45160)
Supplement: Supplementary file 5. — The table shows t-values, degrees of freedom (Dof), p-values and confidence intervals of two-tailed t-tests against zero across subjects for every ROI and dimension (N: number, S: average item size, TFA: total field area) for the number (left table) and size (right table) tasks. [file elife-45160-supp5.docx]

Supplementary File 5.

| Number (N) | | | | | | | | | | | |
| --- | --- | --- | --- | --- | --- | --- | --- | --- | --- | --- | --- |
|  | Task: Judge Number | | | | |  | Task: Judge Size | | | | |
| ROI\stats | t-val | Dof | p-val | CI (95%) | |  | t-val | Dof | p-val | CI (95%) | |
| V1-3 | 8.68 | 19 | <10^-6^ | .22 | .35 |  | 5.25 | 19 | .00004 | .11 | .25 |
| V3AB-V7 | 7.97 | 19 | <10^-6^ | .24 | .41 |  | 4.19 | 19 | .0005 | .06 | .17 |
| IPS 1-5 | 6.59 | 19 | .000003 | .21 | .40 |  | 4.28 | 19 | .0004 | .06 | .18 |
| V1 | 7.30 | 19 | .000001 | .17 | .30 |  | 3.75 | 19 | .001 | .06 | .21 |
| V2 | 7.64 | 19 | <10^-6^ | .19 | .33 |  | 5.42 | 19 | .00003 | .12 | .27 |
| V3 | 6.95 | 19 | .000001 | .19 | .35 |  | 4.40 | 19 | .0003 | .07 | .20 |
| V3AB | 6.05 | 19 | .000008 | .15 | .31 |  | 2.42 | 19 | .026 | .01 | .11 |
| V7 | 6.73 | 19 | .000002 | .20 | .37 |  | 3.51 | 19 | .002 | .03 | .14 |
| IPS12 | 6.73 | 19 | .000002 | .21 | .40 |  | 3.70 | 19 | .001 | .05 | .17 |
| IPS345 | 5.45 | 19 | .000003 | .16 | .36 |  | 3.75 | 19 | .001 | .04 | .14 |
| Average Item Size (S) | | | | | | | | | | | |
|  | Task: Judge Number | | | | |  | Task: Judge Size | | | | |
| ROI\stats | t-val | Dof | p-val | CI (95%) | |  | t-val | Dof | p-val | CI (95%) | |
| V1-3 | 2.38 | 19 | .028 | .00 | .06 |  | 1.29 | 19 | .212 | -.01 | .04 |
| V3AB-V7 | -.04 | 19 | .969 | -.03 | .03 |  | 2.29 | 19 | .034 | .00 | .07 |
| IPS 1-5 | -.49 | 19 | .627 | -.04 | .03 |  | 2.53 | 19 | .020 | .01 | .09 |
| V1 | .85 | 19 | .404 | -.02 | .06 |  | .71 | 19 | .486 | -.02 | .04 |
| V2 | 1.75 | 19 | .096 | .00 | .05 |  | .57 | 19 | .573 | -.02 | .03 |
| V3 | 1.80 | 19 | .088 | .00 | .06 |  | .43 | 19 | .673 | -.02 | .03 |
| V3AB | .50 | 19 | .621 | -.03 | .04 |  | 1.32 | 19 | .203 | -.01 | .04 |
| V7 | -.23 | 19 | .819 | -.04 | .03 |  | 1.47 | 19 | .157 | -.01 | .06 |
| IPS12 | -.42 | 19 | .679 | -.04 | .02 |  | 2.24 | 19 | .037 | .00 | .09 |
| IPS345 | -.73 | 19 | .474 | -.04 | .02 |  | 1.74 | 19 | .097 | -.01 | .07 |
| Total Field Area (TFA) | | | | | | | | | | | |
|  | Task: Judge Number | | | | |  | Task: Judge Size | | | | |
| ROI\stats | t-val | Dof | p-val | CI (95%) | |  | t-val | Dof | p-val | CI (95%) | |
| V1-3 | 7.24 | 19 | <10^-6^ | .23 | .42 |  | 5.44 | 19 | <10^-6^ | .19 | .43 |
| V3AB-V7 | 4.23 | 19 | .00005 | .05 | .16 |  | 3.12 | 19 | .006 | .04 | .19 |
| IPS 1-5 | 2.00 | 19 | .060 | .00 | .11 |  | 1.70 | 19 | .105 | -.01 | .10 |
| V1 | 7.35 | 19 | <10^-6^ | .20 | .36 |  | 6.31 | 19 | <10^-6^ | .19 | .37 |
| V2 | 7.16 | 19 | <10^-6^ | .25 | .46 |  | 5.80 | 19 | <10^-6^ | .20 | .42 |
| V3 | 6.62 | 19 | <10^-6^ | .20 | .38 |  | 5.65 | 19 | <10^-6^ | .19 | .42 |
| V3AB | 4.70 | 19 | .0002 | .08 | .20 |  | 3.60 | 19 | .002 | .06 | .22 |
| V7 | 2.83 | 19 | .011 | .02 | .11 |  | 2.08 | 19 | .051 | .00 | .12 |
| IPS12 | 1.89 | 19 | .074 | -.01 | .10 |  | 1.76 | 19 | .094 | -.01 | .10 |
| IPS345 | 1.43 | 19 | .170 | -.02 | .09 |  | .55 | 19 | .588 | -.04 | .06 |
