# Supplementary material for: Attentional amplification of neural codes for number independent of other quantities along the dorsal visual stream
Source: eLife. 2019 Jul 24;8:e45160. doi: 10.7554/eLife.45160 (PMC6693892; doi:10.7554/eLife.45160)
Supplement: Supplementary file 6. — The table shows t-values, degrees of freedom (Dof), p-values and confidence intervals of two-tailed t-tests against zero across subjects for every ROI and dimension (S: average item size, TFA: total field area, TSA: total surface area, D: density) for the number (left table) and size (right table) tasks. [file elife-45160-supp6.docx]

Supplementary File 6.

| Average Item Size (S) | | | | | | | | | | | |
| --- | --- | --- | --- | --- | --- | --- | --- | --- | --- | --- | --- |
|  | Task: Judge Number | | | | |  | Task: Judge Size | | | | |
| ROI\stats | t-val | Dof | p-val | CI (95%) | |  | t-val | Dof | p-val | CI (95%) | |
| V1-3 | -1.93 | 19 | .069 | -.06 | .00 |  | -2.60 | 19 | .018 | -.06 | -.01 |
| V3AB-V7 | -3.59 | 19 | .002 | -.09 | -.02 |  | .77 | 19 | .449 | -.02 | .05 |
| IPS 1-5 | -2.27 | 19 | .035 | -.08 | .00 |  | 1.38 | 19 | .184 | -.02 | .08 |
| V1 | -1.71 | 19 | .104 | -.07 | .01 |  | -1.11 | 19 | .279 | -.05 | .02 |
| V2 | -2.64 | 19 | .016 | -.06 | -.01 |  | -3.69 | 19 | .002 | -.07 | -.02 |
| V3 | -1.88 | 19 | .075 | -.06 | .00 |  | -2.38 | 19 | .028 | -.05 | .00 |
| V3AB | -2.31 | 19 | .032 | -.07 | .00 |  | -.12 | 19 | .909 | -.03 | .02 |
| V7 | -2.28 | 19 | .034 | -.09 | .00 |  | .74 | 19 | .471 | -.03 | .06 |
| IPS12 | -2.39 | 19 | .028 | -.08 | .00 |  | 1.28 | 19 | .216 | -.02 | .08 |
| IPS345 | -2.29 | 19 | .034 | -.07 | .00 |  | 1.07 | 19 | .298 | -.02 | .07 |
| Total Field Area (TFA) | | | | | | | | | | | |
|  | Task: Judge Number | | | | |  | Task: Judge Size | | | | |
| ROI\stats | t-val | Dof | p-val | CI (95%) | |  | t-val | Dof | p-val | CI (95%) | |
| V1-3 | 6.32 | 19 | <10^-6^ | .17 | .34 |  | 4.96 | 19 | <10^-6^ | .16 | .38 |
| V3AB-V7 | 1.64 | 19 | .118 | -.01 | .10 |  | 2.20 | 19 | .041 | .00 | .16 |
| IPS 1-5 | -1.12 | 19 | .278 | -.08 | .02 |  | .14 | 19 | .892 | -.05 | .06 |
| V1 | 6.30 | 19 | <10^-6^ | .14 | .28 |  | 5.92 | 19 | <10^-6^ | .15 | .32 |
| V2 | 6.33 | 19 | <10^-6^ | .19 | .38 |  | 5.36 | 19 | <10^-6^ | .16 | .37 |
| V3 | 5.70 | 19 | <10^-6^ | .14 | .31 |  | 5.14 | 19 | <10^-6^ | .16 | .39 |
| V3AB | 3.30 | 19 | .004 | .03 | .16 |  | 3.20 | 19 | .005 | .04 | .21 |
| V7 | -.05 | 19 | .958 | -.05 | .05 |  | .98 | 19 | .339 | -.04 | .10 |
| IPS12 | -1.22 | 19 | .239 | -.08 | .02 |  | .22 | 19 | .831 | -.05 | .06 |
| IPS345 | -1.22 | 19 | .238 | -.09 | .02 |  | -.87 | 19 | .397 | -.07 | .03 |
| Total Surface Area (TSA) | | | | | | | | | | | |
|  | Task: Judge Number | | | | |  | Task: Judge Size | | | | |
| ROI\stats | t-val | Dof | p-val | CI (95%) | |  | t-val | Dof | p-val | CI (95%) | |
| V1-3 | 6.21 | 19 | <10^-6^ | .09 | .19 |  | 5.98 | 19 | <10^-6^ | .07 | .15 |
| V3AB-V7 | 4.31 | 19 | .0004 | .05 | .15 |  | 2.82 | 19 | .011 | .01 | .09 |
| IPS 1-5 | 2.50 | 19 | .022 | .01 | .10 |  | 1.93 | 19 | .068 | .00 | .09 |
| V1 | 5.52 | 19 | <10^-6^ | .07 | .15 |  | 4.18 | 19 | .0005 | .04 | .11 |
| V2 | 6.24 | 19 | <10^-6^ | .09 | .18 |  | 5.70 | 19 | <10^-6^ | .08 | .17 |
| V3 | 5.38 | 19 | <10^-6^ | .08 | .17 |  | 4.00 | 19 | .0007 | .04 | .12 |
| V3AB | 3.76 | 19 | .001 | .04 | .14 |  | 1.97 | 19 | .064 | .00 | .07 |
| V7 | 3.06 | 19 | .006 | .02 | .12 |  | 1.68 | 19 | .110 | -.01 | .07 |
| IPS12 | 2.47 | 19 | .023 | .01 | .10 |  | 1.98 | 19 | .062 | .00 | .09 |
| IPS345 | 1.78 | 19 | .091 | -.01 | .08 |  | 1.08 | 19 | .294 | -.02 | .07 |
| Density (D) | | | | | | | | | | | |
|  | Task: Judge Number | | | | |  | Task: Judge Size | | | | |
| ROI\stats | t-val | Dof | p-val | CI (95%) | |  | t-val | Dof | p-val | CI (95%) | |
| V1-3 | 4.63 | 19 | .0002 | .09 | .25 |  | 4.36 | 19 | .00003 | .06 | .16 |
| V3AB-V7 | 4.28 | 19 | .0004 | .07 | .19 |  | 2.48 | 19 | .023 | .01 | .13 |
| IPS 1-5 | 5.11 | 19 | <10^-6^ | .10 | .25 |  | 3.99 | 19 | .0008 | .05 | .17 |
| V1 | 5.27 | 19 | <10^-6^ | .11 | .25 |  | 3.92 | 19 | .0009 | .05 | .18 |
| V2 | 4.39 | 19 | .0003 | .09 | .24 |  | 3.75 | 19 | .001 | .05 | .17 |
| V3 | 3.89 | 19 | .001 | .06 | .20 |  | 2.78 | 19 | .012 | .02 | .12 |
| V3AB | 2.91 | 19 | .009 | .02 | .14 |  | .86 | 19 | .402 | -.03 | .07 |
| V7 | 4.58 | 19 | .0002 | .07 | .19 |  | 2.58 | 19 | .018 | .01 | .13 |
| IPS12 | 5.02 | 19 | <10^-6^ | .10 | .24 |  | 3.49 | 19 | .002 | .04 | .16 |
| IPS345 | 5.08 | 19 | <10^-6^ | .09 | .22 |  | 3.46 | 19 | .003 | .03 | .14 |
