# Supplementary material for: Attentional amplification of neural codes for number independent of other quantities along the dorsal visual stream
Source: eLife. 2019 Jul 24;8:e45160. doi: 10.7554/eLife.45160 (PMC6693892; doi:10.7554/eLife.45160)
Supplement: Transparent reporting form [file elife-45160-transrepform.pdf]

1st Floor
24 Hills Road
Cambridge CB2 1JP, UK

P 01223 855340
W elifesciences.org
T @elife

eLife's transparent reporting form

We encourage authors to provide detailed information *within their submission* to facilitate the interpretation and replication of experiments. Authors can upload supporting documentation to indicate the use of appropriate reporting guidelines for health-related research (see EQUATOR Network), life science research (see the BioSharing Information Resource), or the ARRIVE guidelines for reporting work involving animal research. Where applicable, authors should refer to any relevant reporting standards documents in this form.

If you have any questions, please consult our Journal Policies and/or contact us: editorial@elifesciences.org.

Sample-size estimation

- You should state whether an appropriate sample size was computed when the study was being designed
- You should state the statistical method of sample size computation and any required assumptions
- If no explicit power analysis was used, you should describe how you decided what sample (replicate) size (number) to use

Please outline where this information can be found within the submission (e.g., sections or figure legends), or explain why this information doesn't apply to your submission:

Sample size was not specifically estimated prior to the study, however it was equal or larger than the one typically used in experiments in the field, see the beginning of the Methods section (Subject and MRI acquisition paragraph, page 16) for a reference list of previously published experiments with similar sample size.

Replicates

- You should report how often each experiment was performed
- You should include a definition of biological versus technical replication
- The data obtained should be provided and sufficient information should be provided to indicate the number of independent biological and/or technical replicates
- If you encountered any outliers, you should describe how these were handled
- Criteria for exclusion/inclusion of data should be clearly stated
- High-throughput sequence data should be uploaded before submission, with a private link for reviewers provided (these are available from both GEO and ArrayExpress)

Please outline where this information can be found within the submission (e.g., sections or figure legends), or explain why this information doesn't apply to your submission:

In this study we collected fMRI data on human adult subjects (N=20, which corresponds to a biological replicate). Each participant performed the entire experiment once, within which different experimental conditions were presented in a randomized order. We provided information about the inclusion criteria (only normal or corrected to normal vision) in the manuscript (see page 16, first line of Methods section) and we did not exclude any participant from the study after data acquisition.

Statistical reporting

- Statistical analysis methods should be described and justified

eLife Sciences Publications, Ltd is a limited liability non-profit non-stock corporation incorporated in the State of Delaware, USA, with company number 5030732, and is registered in the UK with company number FC030576 and branch number BR015634 at the address 1st Floor, 24 Hills Road, Cambridge CB2 1JP | August 2014

- Raw data should be presented in figures whenever informative to do so (typically when N per group is less than 10)
- For each experiment, you should identify the statistical tests used, exact values of N, definitions of center, methods of multiple test correction, and dispersion and precision measures (e.g., mean, median, SD, SEM, confidence intervals; and, for the major substantive results, a measure of effect size (e.g., Pearson's r, Cohen's d)
- Report exact p-values wherever possible alongside the summary statistics and 95% confidence intervals. These should be reported for all key questions and not only when the p-value is less than 0.05.

Please outline where this information can be found within the submission (e.g., sections or figure legends), or explain why this information doesn't apply to your submission:

Statistical analysis methods are described in the 'Image Processing and Data Analysis' paragraph in the Methods section. Exact p-values (unless smaller than $<10^{-6}$) alongside with degrees of freedom and F or t values are reported in the Results section, and in Supplementary Files. While plots show mean values across subjects and SEM, we provided corresponding individual subjects values as source data.

(For large datasets, or papers with a very large number of statistical tests, you may upload a single table file with tests, Ns, etc., with reference to sections in the manuscript.)

Group allocation

- Indicate how samples were allocated into experimental groups (in the case of clinical studies, please specify allocation to treatment method); if randomization was used, please also state if restricted randomization was applied
- Indicate if masking was used during group allocation, data collection and/or data analysis

Please outline where this information can be found within the submission (e.g., sections or figure legends), or explain why this information doesn't apply to your submission:

This is a study in a single group of subjects. All experimental manipulations were within subject.

Additional data files ("source data")

- We encourage you to upload relevant additional data files, such as numerical data that are represented as a graph in a figure, or as a summary table
- Where provided, these should be in the most useful format, and they can be uploaded as "Source data" files linked to a main figure or table
- Include model definition files including the full list of parameters used
- Include code used for data analysis (e.g., R, MatLab)
- Avoid stating that data files are "available upon request"

Please indicate the figures or tables for which source data files have been provided:

eLife Sciences Publications, Ltd is a limited liability non-profit non-stock corporation incorporated in the State of Delaware, USA, with company number 5030732, and is registered in the UK with company number FC030576 and branch number BR015634 at the address 1st Floor, 24 Hills Road, Cambridge CB2 1JP | August 2014

eLIFE
1st Floor
24 Hills Road
Cambridge CB2 1JP, UK
P 01223 855340
W elifesciences.org
T @elife

Individual subjects' data points for behavioural and fMRI results for all regions of interest, corresponding to Figures 2A, 3C, 5, Figure 3—figure supplements 1 and 2, and Figure 5—figure supplements 1 and 2 as. cvs files. The maps displayed in figure 2B-D and 3B are provided in a format readable with Freesurfer/Freeview, one of the most widely used free neuroimaging softwares. The functional imaging dataset is available via the Open Science Framework (https://osf.io/6zch2, Open Science Framework, 6zch2, archive, Open Science Framework).
